# LGBTQ+ Couples’ Sexual Health Experiences During Pregnancy and Postpartum: A Qualitative Survey Study

**DOI:** 10.1080/19317611.2026.2643656

**Published:** 2026-03-16

**Authors:** Anna Malmquist, Ellen Norén, Katri Nieminen, Hanna Grundström

**Affiliations:** a Department of Behavioural Sciences and Learning, Linköping University, Linköping, Sweden; b Department of Obstetrics and Gynaecology in Norrköping, Norrköping, Sweden; c Department of Biomedical and Clinical Sciences, Linköping University, Linköping, Sweden; d Department of Health, Medicine and Caring Sciences, Linköping University, Linköping, Sweden

**Keywords:** Sexual and gender minority, sexual function, sex drive, same-sex intimacy, transgender

## Abstract

**Background:**

Sexual health is often negatively affected during pregnancy and the postpartum period in the general pregnant and birthing population. Although an increasing number of LGBTQ+ individuals are becoming parents, their experiences of sex during pregnancy and postpartum have not been studied.

**Aim:**

To explore whether, and in what ways, sex life was affected during pregnancy and postpartum among LGBTQ+ couples and individuals.

**Methods:**

LGBTQ+ pregnant individuals (*n* = 301) and partners of pregnant individuals (*n* = 104) responded to a digital survey during pregnancy, with a postpartum follow-up response rate of 74.3%. Free-text responses (*n* = 526) describing experiences of sex life were analyzed using codebook thematic analysis.

**Findings and discussion:**

A decrease in sexual desire was common and was generally associated with physical complications and mental stress. Intimacy was often maintained without sexual activity. A lack of LGBTQ+ inclusive information on safe sex during pregnancy and postpartum was noted. Active engagement by healthcare staff in addressing sexuality during these periods is essential for providing relevant and LGBTQ+ specific information.

**Conclusion:**

Specific knowledge of LGBTQ+ sexual practices among healthcare staff is important, as are affirming attitudes toward less normative sexual practices.

## STATEMENT OF SIGNIFICANCE

*Problem or issue:* Despite more LGBTQ+ individuals becoming parents, no research has examined their sexual health during pregnancy and postpartum.

*What is already known:* Sexual health is often negatively affected during pregnancy and the postpartum period in the general population. Some LGBTQ+ individuals fear disclosing their sexual activities, which lowers the likelihood of receiving appropriate care.

*What this paper adds:* A decrease in sexual desire was common among participants and generally associated with physical complications and mental stress. Intimacy was often maintained without sexual activity. A lack of LGBTQ+ inclusive information on safe sex during pregnancy and postpartum was noted.

## Introduction

Sexual health is, for most people, an essential component of physical and emotional well-being (World Health Organization: Department of Reproductive Health and Research, 2010), but it is often affected during pregnancy and the postpartum period, commonly through reduced sexual desire, function, and activity (Dawson, Vaillancourt-Morel, et al., 2020; Fernández-Carrasco et al., 2023; Gałązka et al., 2015; Serati et al., 2010). When depression occurs during pregnancy and/or postpartum, sexual desire is often negatively affected, and the risk of relationship problems increases (Johnson, 2011). Traumatic birth experiences may also contribute to relationship distress and negatively affect sex life (Garthus-Niegel et al., 2018).

Opportunities for lesbian, gay, bisexual, transgender, queer, and other gender and sexual minority (LGBTQ+) individuals to become parents have increased in recent decades, leading to more LGBTQ+ individuals experiencing pregnancy and birth (Greenfield et al., 2025). General research on the sexual health of LGBTQ+ individuals has shown that stigma and insufficient competence among healthcare professionals can be problematic (Jennings et al., 2019; Taylor & King, 2021). However, no previous research has *specifically* focused on this group’s sexual health during pregnancy and in the postpartum period.

### Sexual health during pregnancy

In studies of general pregnant populations, predominantly consisting of heterosexual cisgender women, a decrease in sexual health is commonly reported (Fernández-Carrasco et al., 2023). Sexual desire, function, and activity tend to fluctuate during pregnancy, typically peaking in the second trimester and being lower in the first and third trimesters. An early decline in sexual desire is often related to physical discomfort, although mental stress can also play a role. During the second trimester, sexual desire may increase with improved physical well-being, whereas a decrease in the third trimester is common due to physical limitations associated with the growing belly and concerns about harming the baby or the pregnant individual.

Some prospective parents fear that sexual activity may cause miscarriage or induce labor (de Pierrepont et al., 2022). Such risks are rare and are usually based on misconceptions. Lack of information about sex during pregnancy is a common reason for avoiding sexual activity (Serati et al., 2010). Nevertheless, the majority of heterosexual couples remain sexually active until the seventh month, and one-third continue until the ninth month (Lehmiller et al., 2014). Body image can also influence sexual health during pregnancy, as negative feelings about bodily changes are associated with lower levels of sexual desire and satisfaction (Chang et al., 2011).

Research on the sexual health of pregnant individuals’ partners is relatively limited. Studies of general populations, in which most partners are heterosexual cisgender men, indicate that sexual desire gradually decreases as pregnancy progresses, reaching its lowest point during the third trimester (Fernández-Carrasco et al., 2023). Fear of witnessing the upcoming birth, causing harm during intercourse, or anxiety about impending fatherhood has been associated with decreased sexual desire (Johnson, 2011). However, the decline in desire is generally less pronounced in the non-pregnant partner compared to the pregnant partner (Fernández-Carrasco et al., 2023).

### Sexual health postpartum

Research on general birthing populations indicates that sexual problems are common 3–6 months postpartum, although generally declining over time (Dawson, Vaillancourt-Morel, et al., 2020; de Judicibus & McCabe, 2002; Serati et al., 2010). Pain during intercourse and pelvic floor dysfunction commonly delay the resumption of sexual activity (Serati et al., 2010). In addition, breastfeeding may cause vaginal dryness (Johnson, 2011). While the majority of heterosexual couples resume sexual activity within three months postpartum, the woman’s first experience is often not particularly pleasurable and is usually initiated by the partner (Gillen et al., 2025). A healthy body image and low self-criticism are associated with greater sexual pleasure and an earlier resumption of sexual activity (Gillen et al., 2025; Ozcan, 2024).

Sexual health can also be affected in the non-birthing partner postpartum (Gungor et al., 2008; Johnson, 2011; van Anders et al., 2013). The transition to parenthood may alter the relationship dynamic, which in turn can affect the couple’s shared sexual life.

### LGBTQ+ sexual health

Some research has documented poorer sexual health among LGBTQ+ individuals, highlighting how discrimination and normative attitudes within the healthcare system can lead to delayed or avoided healthcare-seeking when problems arise (Jennings et al., 2019; Lee & Kanji, 2017; Silveri et al., 2022; Taylor & King, 2021). However, Borneskog et al. (2014a) found higher sexual satisfaction among the lesbian compared to heterosexual couples before the onset of IVF treatment and three years later.

Some LGBTQ+ individuals fear disclosing their sexual activities in healthcare contacts, which in turn lowers the likelihood of receiving appropriate care (Taylor & King, 2021).

### Transition to parenthood among LGBTQ+ individuals

Opportunities for LGBTQ+ individuals to achieve parenthood have increased over the past decades, with technological advances and legal reforms making assisted reproduction accessible to a more diverse population (Greenfield et al., 2025). Some studies indicate elevated rates of mental ill-health in this group during the transition to parenthood (Gonzales et al., 2019; Hartnett et al., 2021), while other studies report better health (Borneskog et al., 2014b). Negative experiences in healthcare during pregnancy and birth may induce minority stress (Klittmark et al., 2023; Malmquist et al., 2019) and contribute to reproductive injustice (Klittmark et al., 2024).

Despite previous studies reporting lower general sexual health among LGBTQ+ individuals (Taylor & King, 2021) and others indicating elevated rates of mental ill-health in this group during the transition to parenthood (Gonzales et al., 2019; Hartnett et al., 2021), no research has specifically examined LGBTQ+ individuals’ sexual health during pregnancy and the postpartum period. The aim of the present study was therefore to explore whether, and in what ways, sex life was affected during pregnancy and postpartum among LGBTQ+ couples and individuals.

## Methods

### Study design and data collection

This study is part of a longitudinal project examining the well-being and experiences of LGBTQ+ individuals during pregnancy and postpartum, approved by the Swedish Ethical Review Authority (2021-05801-01 and 2023-07128-02). Participants were recruited via social media advertisements and antenatal clinic posters using convenience sampling. These advertisements included a web link and QR code for a digital survey. Before accessing the survey itself, participants were presented with a page describing the study aim and procedures. They were also informed that participation was voluntary and uncompensated. Providing that they met the eligibility criteria—being over 18 years of age and expecting a child as either the pregnant or non-pregnant prospective parent—participants gave their informed consent to participate and were invited to complete the survey. Data were collected from March 2022 to September 2025. Participants completed digital surveys during pregnancy (at gestational week ≥25) and postpartum (sent out 6–8 weeks after the expected due date). While the risks of participation were generally considered low, participants were informed that responding to questions about one’s pregnancy and birth experiences may cause stress. In case any participant was in need of support, they were provided with contact details for an LGBTQ+ competent midwife, independent of the research group. All survey questions were carefully designed to be LGBTQ+ inclusive to minimize the risk of causing minority stress. Beyond contributing to increased knowledge about LGBTQ+ individuals’ health and care needs during the transition to parenthood, there were no direct benefits for individual participants.

Although both pregnant and non-pregnant prospective parents were invited to participate, each participant responded individually. If both partners in a couple chose to participate, no questions were included that would allow the researchers to identify them as belonging to the same couple. Efforts were made to reach non-pregnant prospective parents through targeted advertising; however, this group was underrepresented in the final sample.

Participants were asked whether pregnancy and birth had affected their sexual life, with response options of “a lot,” “somewhat,” or “not at all.” For those selecting “a lot” or “somewhat,” an open-ended prompt followed, asking them to describe how their sexual life had been affected (Table 1). These free-text responses were selected for the analysis. In total, 526 free-text responses were analyzed.

**Table 1.** Main and follow-up questions in the survey.

	Question during pregnancy	Question postpartum	Follow-up question
Questions for birthing parent	Has your sexual life been affected by the pregnancy?	Has your sexual life been affected by the pregnancy and/or the birth?	Would you like to describe how your sexual life has been affected? Would you like to describe how your sexual life has been affected?
Questions for non-birthing parent	If you are in a sexually intimate relationship with the birthing parent, has your sexual life been affected by the pregnancy?	If you are in a sexually intimate relationship with the birthing parent, has your sexual life been affected by the pregnancy and/or the birth?	

### Participants

During pregnancy, 405 participants responded, of whom 301 (74.3%) also completed the postpartum survey. Among the participants, 74.3% were birthing parents and 25.7% were non-birthing parents.

Birthing parents (*n* = 301) were aged 22–44 years (mean = 32.3) and completed the survey during gestational weeks 25–41 and 3–32 weeks postpartum. Most were cisgender women (92.4%), 5.6% nonbinary, and 1.3% transgender men. Half identified as bi/pansexual (50.5%) and 44.9% as lesbian. Non-birthing parents (*n* = 104) were aged 25–47 years (mean = 33.6) and completed the survey when their partners were in gestational weeks 25–42 and 3–25 weeks postpartum. Most were cisgender women (80.8%), 6.7% transgender men, 6.7% nonbinary, 4.8% cisgender men, and 1.0% transgender women. They were primarily lesbian (58.7%) or bi/pansexual (28.8%). More sociodemographic details are found in Table 2.

**Table 2.** Sociodemographic characteristics.

Variables	Birthing parents (*n* = 301)	Non-birthing parents (*n* = 104)
**Age, span (*m*)**	22–44 (32.3)	25–47 (33.6)
**Gestational week, span (*M*)**	25–41 (29)	25–42 (30)
**Postpartum week, span (*M*)**	3–32 (8)	3–25 (10)
**Gender identity, *n* (%)**		
Woman (cis)	278 (92.4)	84 (80.8)
Nonbinary/genderqueer	17 (5.6)	7 (6.7)
Man (trans)	4 (1.3)	7 (6.7)
Man (cis)	–	5 (4.8)
Woman (trans)	–	1 (1.0)
Other	2 (0.7)	–
**Sexual identity, *n* (%)**		
Lesbian	135 (44.9)	61(58.7)
Bi/pansexual	152 (50.5)	30 (28.8)
Queer	7 (2.3)	7 (6.7)
Heterosexual	–	3 (2.9)
Asexual	3 (1.0)	1 (1.0)
Other	4 (1.3)	2 (1.9)
**Residential area, *n* (%)**		
Major city	204 (67.8)	79 (76.0)
Small town	53 (17.6)	15 (14.4)
Countryside	43 (14.3)	8 (7.7)
Missed response	1 (0.3)	2 (1.9)
**Place of birth, *n* (%)**		
Sweden	273 (90.7)	96 (92.3)
Other Nordic country	7 (2.3)	2 (1.9)
Other European country	10 (3.3)	3 (2.9)
Outside Europe	10 (3.3)	2 (1.9)
Missed response	1 (0.3)	1 (1.0)
**Relationship status, *n* (%)**		
Married/co-habiting	274 (91.0)	103 (99.0)
Single	14 (4.7)	–
Partner living apart	10 (3.3)	–
Multiple partners	3 (1.0)	1 (1.0)
**Educational level, *n* (%)**		
University	256 (85.0)	92 (88.5)
Highschool	38 (12.6)	9 (86.5)
Mandatory school	2 (0.7)	–
Other	5 (1.7)	3 (2.9)
**Occupation during pregnancy, *n* (%)**		
Full-time employed	216 (71.8)	79 (76.0)
Part-time employed	42 (14.0)	10 (9.6)
Studying	20 (6.6)	9 (8.7)
Longterm sick leave	10 (3.3)	5 (4.8)
Parental leave	8 (2.7)	–
Unemployed	5 (1.7)	1 (1.0)
**Conception method, *n* (%)**		
IVF	150 (49.9)	59 (56.7)
Insemination at clinic	71 (23.6)	41 (39.4)
Intercourse	70 (23.3)	3 (2.9)
Self-insemination	10 (3.3)	1 (1.0)

### Data analysis

Codebook Thematic Analysis (TA) is a structured method for analyzing qualitative data (Braun & Clark, 2021, 2022a). It employs a predefined codebook—a set of labels or “codes”—to organize data into themes. Compared to reflexive TA, Codebook TA is more structured and less open-ended.

Initially, the transcripts were read thoroughly several times to gain familiarity with the material, during which relevant sections were highlighted and compiled separately. While familiarizing themselves with the data, the researchers also developed a priori themes based on previous research and their preunderstanding (Braun & Clark, 2022b). In the subsequent phases, the excerpts were systematically coded in detail. Codes were grouped into potential overarching themes, with related data extracts compiled in a codebook. A thematic map was created to illustrate relationships between themes. By the end of this phase, themes, subthemes, and extracts were fully organized within the codebook. In the final stages, the themes were clearly defined and named, illustrative quotes were selected, and the findings were compiled into the written results. Six main themes were constructed during the analysis.

## Findings and discussion

The majority of participants reported that pregnancy had impacted their sex life, with an even higher proportion reporting an impact during the postpartum period (see Table 3).

**Table 3.** Reported pregnancy and birth’s affection on sexual life, and used free-text prompts.

During pregnancy, *n* (%)	Pregnant parents (*n* = 301)	Non-pregnant parents (*n* = 104)
Affected a lot	80 (26.6)	32 (30.8)
*Free-text response*	*79 (98.8)*	*28 (87.5)*
Somewhat affected	166 (55.1)	52 (50.0)
*Free-text response*	*151 (91.0)*	*39 (75.0)*
Not at all affected	55 (18.3)	19 (18.3)
*Free-text response*	*Not prompted*	*Not prompted*
No sexual relationship with pregnant person	Not optional	1 (1.0)
*Free-text response*	–	*Not prompted*
Postpartum, *n* (%)	Birthing parents (*n* = 225)	Non-birthing parents (*n* = 76)
Affected a lot	93 (41.3)	41 (53.9)
*Free-text response*	*88 (94.6)*	*37 (90.2)*
Somewhat affected	94 (41.8)	28 (36.8)
*Free-text response*	*80 (85.1)*	*24 (85.7)*
Not at all affected	38 (16.9)	6 (7.9)
*Free-text response*	*Not prompted*	*Not prompted*
No sexual relationship with pregnant person	Not optional	1 (1.3)
*Free-text response*	–	*Not prompted*

Below, the analysis of free-text responses is presented and interpreted in relation to previous research. The first section focuses on the impact on sex life during pregnancy, while the second addresses the postpartum period. Each section includes three themes (see Table 4). All quotes are accompanied by the participant’s sexual and gender identities, age (e.g., 31y), gestational age (e.g., 29w) or time since birth (e.g., 8w), and their current role as birthing parent (BP) or non-birthing parent (NBP).

**Table 4.** Themes.

	Themes
Experiencing sex life during pregnancy	The desire is not the same
	Physical or mental ill-health hinders
	Taboos, lack of information, and low self-confidence as mental blockers
Experiencing sex life postpartum	Birth complications and trauma hinder
	Settling as parents is in focus
	The return of desire

### Experiencing sex life during pregnancy

#### The desire is not the same

Most pregnant participants experienced a decrease in sexual desire, which in many cases led to a decline in sexual activity. Many participants provided brief statements about the decline in desire, such as: “Not as interested in sex when pregnant” (lesbian cisgender woman, 39y/27w, BP), while others elaborated further on their experiences:

Less frequent, but it’s not something either of us sees as a problem. We just both seem to have a lower desire in general right now (lesbian nonbinary, 35y/27w, NBP)

This participant described a decrease in sexual activity resulting from both partners’ reduced desire, noting that they appeared to be aligned emotionally and approached the change with an attitude of acceptance.

Although sexual desire and activity had declined, both pregnant and non-pregnant participants emphasized that other forms of closeness and intimacy had increased. They described various ways of expressing affection and tenderness:

I don’t want to have sex. I feel uncomfortable with that kind of intimacy. We’ve only had sex three times since I got pregnant. Still, we maintain closeness and affection between us. But sexuality feels almost repulsive (bisexual cisgender woman, 36y/26w, BP)

While the excerpt reveals a strong sense of discomfort associated with sex, this was contrasted by a maintained sense of intimacy and affection, suggesting that the couple had been able to manage the experience well. Another participant described the shift from sexual activity to other forms of closeness as natural:

No sex. But we continue with physical touch, hugs, etc. It feels natural as our bodies, hormones, etc. change during/after pregnancy. (lesbian cisgender woman, 29y/26w, NBP)

The participant suggested that her partner’s change in desire was a natural consequence of the physical changes during pregnancy, reflecting an accepting approach toward replacing sexual activity with other forms of closeness. In contrast, a few participants reported that they did not desire any physical closeness, which in some cases negatively affected the couple’s relationship. While it is well established that a decline in sexual desire is common during pregnancy (Fernández-Carrasco et al., 2023), these findings offer a nuanced understanding of how such declines are experienced and navigated among LGBTQ+ couples.

In contrast to the common experience of decreased desire, some participants described fluctuating desire, as illustrated by this participant: “Less and higher sex lust, fluctuating during pregnancy” (queer cisgender woman, 41y/34w, BP). A more general increase in sexual desire was also reported by some participants:

We have sex more often. I also feel more comfortable with my body now during pregnancy than I did before. I’m able to receive appreciation in a different way, which feels nice. Also find myself wanting closeness more than before, and my partner responds to that. (bisexual cisgender woman, 27y/25w, BP)

The excerpt illustrates an increase in sexual desire and activity, alongside a sense of comfort with the pregnant body. This reflects how some individuals feel more at ease during pregnancy, which in turn may enhance the desire for closeness and sex.

#### Physical or mental ill-health hinders

Both pregnant and non-pregnant participants commonly reported that physical discomfort and somatic complications during pregnancy hindered sexual activity, similar to findings from studies on general pregnant populations (Fernández-Carrasco et al., 2023). Participants described bleeding, pain, nausea, uncomfortable contractions, lack of lubrication, a sense of clumsiness, and changes in sensation. One participant shared her struggles with nausea:

Due to extreme nausea, we didn’t have sex at all during the first 3–4 months of the pregnancy (lesbian cisgender woman, 22y/37w, BP).

A non-pregnant participant also described how his partner’s pregnancy complications led to a decline in sexual activity:

Due to pelvic instability and pain issues my wife has been experiencing, our sexual relationship has cooled down a bit. Which feels reasonable given the circumstances (bisexual, polyamorous cisgender man, 39y/28w, NBP)

Notably, it seems important for some of the non-pregnant partners to stress that they too are okay with the absence of sex and do not wish to exert any pressure on their partners.

Besides somatic complications, several participants reported a decrease in sexual desire and activity due to mental stress or ill health. The negative impact of mental ill health on relationship quality is well documented in research on general pregnant populations (Garthus-Niegel et al., 2018), as is the increased risk of depression during pregnancy in both prospective parents (Aziz et al., 2025; Rao et al., 2020). Participants in the present study mentioned various stressors, such as depression, burnout syndrome, grief, and difficulties in finding time and energy. One participant described the mental ill health her wife experienced:

During the second trimester, I’ve experienced an increased sex drive despite the fatigue. However, my wife has also been extremely tired and worn out from taking on extra responsibility when I haven’t had the energy. So, our sex life together hasn’t been very active, unfortunately. (lesbian cisgender woman, 36y/28w, BP)

The quote illustrates how both partners were struggling with a lack of energy, which negatively impacted their sex life. For some, mental ill health also affected emotional closeness: “Mental health struggles have caused our relationship to drift apart.” (bisexual cisgender woman, 27y/35w, BP). In such cases, relationship difficulties appear to have influenced their sex life.

Some participants further pointed to side effects of SSRIs and other medications: “Less sex, due to medication and other factors, so the desire hasn’t always been there for my partner.” (bisexual transgender man, 32y/39w, NBP). One specific stressor mentioned by a few participants was related to the process of becoming pregnant:

The main impact was during the IVF process, which has also affected things during the pregnancy. All the medication and the pressure during the process made me lose almost all desire for sex, both physically and mentally. (lesbian cisgender woman, 39y/26w, BP)

Stress and pressure associated with the IVF process led to a loss of sexual desire for this couple, which persisted during the subsequent pregnancy.

#### Taboos, lack of information, and low self-confidence as mental blockers

In addition to difficulties related to physical changes, the unborn baby occupied mental space for the prospective parents. Both pregnant and non-pregnant participants mentioned feeling uncomfortable having sex with a baby in the womb, which negatively affected their ability to relax and enjoy sex. One pregnant participant described:

It [sex] barely exists, but mostly because of my own feelings of sensing a child within me while something sexual is happening, it just mentally shuts down for me. (lesbian cisgender woman, 30y/37w, BP)

The quote highlights how sex during pregnancy may evoke an incest taboo. Another participant described a different taboo, mentioning that they “haven’t felt completely comfortable, for example, at a sex club while visibly pregnant.” (bisexual and polyamorous nonbinary, 34y/33w, BP). While the participant did not elaborate on why they felt uncomfortable at sex clubs, one possible interpretation is that the pregnant body’s strong connotations to family life conflict with certain forms of sexual activity—in this case, when sex is performed in front of others and/or with more than one partner. Both sex and pregnancy are surrounded by strong social norms, stipulating what is considered, for example, normal, dignified, queer, or deviant (Greenfield et al., 2025). The intersection of pregnancy and sex carries its own unique normativity, in which pregnancy is associated with more normative sexual practices, while less normative activities may be considered appropriate only for non-pregnant individuals.

Another factor negatively affecting sex during pregnancy was a fear of physically harming the baby in the womb, such as causing miscarriage or inducing preterm birth. Some participants lacked information about which sexual activities are considered safe during pregnancy. One participant explained:

I had a bleeding and an increased risk of miscarriage early in the pregnancy. Everything is fine now, but I haven’t really dared to have sex because of it. No doctor or midwife has asked about my sex life during the pregnancy, and I haven’t really felt like bringing it up myself either. So I haven’t really known whether it’s OK to have sex or not. (bisexual cisgender woman, 33y/25w, BP)

While fear of causing harm to the unborn baby during sex is common (de Pierrepont et al., 2022), such risks are generally low, highlighting the importance of correct and reliable information (Serati et al., 2010). The participant’s reluctance to raise her uncertainties underscores the need for healthcare professionals to possess LGBTQ+ relevant knowledge about sex during pregnancy and to actively address the topic. Minority stress may further contribute to the reluctance to discuss LGBTQ+ sexual matters with midwives, as fear of being met with disregard or ignorance is common among pregnant LGBTQ+ individuals (Malmquist, 2016). Previous research shows that pregnant LGBTQ+ individuals often find midwives kind and accommodating but lacking knowledge about the unique needs of LGBTQ+ individuals during the transition to parenthood (Malmquist et al., 2024). As lack of competence regarding LGBTQ+ sexual practices is commonly known to affect LGBTQ+ individuals’ sexual health in general (Taylor & King, 2021), adequate knowledge of LGBTQ+ sexual practices is particularly important in pregnancy healthcare.

In addition, another participant described:” I’m also afraid of hurting the baby because everything that’s written about is hetero sex during pregnancy, so I don’t really know what’s safe.” (lesbian cisgender woman, 34y/25w, BP). Seeking written information may be a preferred option for those who are not comfortable addressing their concerns about sex in personal contact with healthcare staff, but in this case, the participant did not find LGBTQ+ relevant information. With pregnancy literature predominantly focusing on heterosexual practices, the participant was left unsure whether her own sexual practices might harm the baby. In the absence of such information, LGBTQ+ couples may experience uncertainty, leading them either to (unnecessarily) refrain from sex or to remain anxious about the safety of the sexual activity they do engage in.

Several participants mentioned a feeling of not being physically comfortable or not feeling attractive in their pregnant body. Weight gain, gender dysphoria, and more general feelings of discomfort were described as mental barriers to sexual activity. One participant explained:

Over the past few weeks, I’ve started feeling less agile and not really interested in sex because of that. I also don’t feel attractive due to how my body has changed during the pregnancy. (lesbian cisgender woman, 30y/26w, BP)

While several pregnant participants described feelings of lost attractiveness, none of the non-pregnant participants reported a loss of attraction to their partner; rather, they also emphasized that such feelings belonged to the pregnant partner: “My wife also has no sex drive when she’s pregnant and doesn’t feel comfortable in her body.” (lesbian cisgender woman, 36y/32w, NBP).

Some transgender men and nonbinary individuals mentioned increased gender dysphoria due to pregnancy-related bodily changes, which in turn affected their sex life:

Gender dysphoria has increased during the pregnancy, which has made it difficult for me to be present in my body, as a result, decreased sexual activity with partner and on my own. (bisexual transgender man, 31y/29w, BP)

The participant described that both solo and partnered sexual activity had decreased, suggesting that the experience was not only related to being observed by the partner, but rather reflected an internal stress that hindered even masturbation. Transgender men and nonbinary individuals have previously been identified as a vulnerable group during pregnancy (Falck et al., 2024). It is well established that gender dysphoria, as well as cisnormative attitudes, may cause stress for the pregnant person. What this study adds is that sexual life is specifically affected in this group, as bodily changes due to pregnancy and childbirth may lead to decreased sexual desire and a reduced ability to relax and enjoy sex.

### Experiencing sex life in the postpartum period

#### Birth complications and trauma hinder

Birthing and non-birthing participants commonly reported difficulties engaging in sexual activity during the postpartum period, when the birthing partner’s body had not yet fully healed. Some participants were also experiencing mental health issues and reduced well-being postpartum, which negatively affected sexual desire. These findings correspond closely with previous results from general birthing populations (Dawson, Strickland, et al., 2020; Serati et al., 2010).

Birth-related genital complications, such as pain, bleeding, increased or decreased sensitivity, or general discomfort in the genital area, were commonly reported by participants.

Since I was stitched and cut because of vacuum extraction, it took some time before I felt ready to have sex, and I don’t have as much sex drive as before. (lesbian cisgender woman, 33y/8w, BP)

Some described a lack of information regarding which sexual activities would be considered safe for their genitals, as one participant explained: “The uncertainty makes it difficult to relax and enjoy vaginal penetration. I’m awaiting a visit to the midwife.” (bisexual nonbinary, 39y/9w, BP). Likewise, a non-birthing participant described her uncertainties:” It has felt a bit scary because of the healing, and leave it up to my partner to take the initiative.” (queer cisgender woman, 33y/11w, NBP). Postponing sexual activity felt like the safer option for these participants. It is possible that earlier access to information about safe sexual practices would have reduced their feelings of insecurity.

Some participants, both birthing and non-birthing, explained how mental ill health impeded sexual desire. In line with previous research (Bai et al., 2023; Stén et al., 2023), they mentioned depression and trauma, as well as more general reduced well-being. One participant reported mental ill health in both partners:” The depression has affected […] and now we both take SSRIs, which also impacts libido” (lesbian cisgender woman, 33y/12w, NBP). Another participant had experienced birth trauma:

It feels more difficult with intimacy after my physical boundaries were crossed during birth. It feels as though the birth was an assault, and it affects my sexual life. (lesbian nonbinary, 26y/12w, BP)

The participant was affected by the birth trauma, which caused significant psychological distress and, in turn, negatively affected their sex life.

Some birthing parents reported low sexual self-esteem or feeling unattractive. One participant described:” During pregnancy and postpartum, my self-confidence has been low, which has impacted sex drive.” (bisexual cisgender woman, 33y/8w, BP).

As during pregnancy, transgender men and nonbinary individuals mentioned gender dysphoria as negatively affecting sexual activity in the postpartum period:

Not as frequent sex, and it’s more of a ‘quickie’ and routine. I don’t feel connected to my sexual side. Gender dysphoria has increased around this because I haven’t been able to exercise, and I’m also using my breasts for breastfeeding, etc., and can’t use a binder or other things the same way as before. (queer nonbinary, 34y/9w, BP)

While this participant described continued sexual activity, their desire had declined. In addition, gender dysphoria increased when they were unable to work out or wear a chest binder—strategies they had previously used to minimize dysphoric feelings. Breast/chestfeeding is known to cause particular stress in trans masculine individuals (MacDonald et al., 2016), but its effects on sexual activity have not previously been studied.

#### Settling as parents is in focus

Several participants reported feeling overwhelmed by the transition to parenthood, with caring for the newborn as their main priority. During this period, sexual activity was of the lowest priority. Some expressed anxiety about how to reconnect after an extended period without sexual intimacy. Many described feeling disconnected from their sexual side after childbirth:

Haven’t had the space to think of my body as sexual while also trying to relate to it as a life-giving and breastfeeding body. (queer nonbinary, 34y/9w, BP).

Several birthing parents reported that the constant presence of the baby was the main obstacle to sexual activity. As the baby took priority, time and energy for intimacy were reduced. Many mentioned sleepless nights, lack of energy, and exhaustion, which made it difficult for their sexual life to resume. Some participants considered the baby’s around-the-clock needs stressful and demanding: “I also had a colicky baby who was latched onto my breast and attached to my body 24/7, so I longed to have my body to myself.” (bisexual cisgender woman, 41y/7w, BP). While some participants reported that their focus on the child had negatively affected emotional closeness, many others emphasized that the absence of sexual activity during the postpartum period was expected and felt unproblematic:

We haven’t had the same opportunities for intimacy because of newborn and two-year-old at home, which was very expected. However, I wouldn’t say that it has negatively affected us. (bisexual cisgender woman, 32y/8w, BP)

With this accepting attitude, many couples appeared maintaining closeness and a positive relationship. In contrast to research on heterosexual couples, which has shown that the first sexual experience after childbirth is often partner-initiated and not very pleasurable for the birthing person (Gillen et al., 2025), the present study instead shows how many postponed sexual activity while healing and focused on settling into parenthood, with non-birthing partners waiting for the birthing partner to initiate sexual activity when ready.

#### The return of desire

In contrast to the many accounts of diminished desire and absence of sexual activity, some participants described increased libido and a return of sexual desire after childbirth. Both birthing and non-birthing participants described adapting sexual activity to the current situation, taking into account the healing process of the birthing partner and the needs of the baby. Others expressed hopes for more opportunities in the future and described alternative ways to maintain closeness. Some participants described adjusting when and how to engage in sexual activity during the postpartum period:

After the birth, my sex drive has increased a lot, but we’re waiting until the postnatal check-up for penetrative sex because of my C-section. Mutual touching and masturbation occur however. (bisexual cisgender woman, 33y/6w, BP)

The excerpt reflects an adjustment of sexual practices, postponing vaginal penetration in favor of other forms of sexual activity.

In contrast to participants who expressed an accepting attitude toward the absence of sexual activity, others described the resumption of sexual activity as an active and ongoing process:

Much less sex during the pregnancy and none now afterwards. We talk about it and look forward to when my partner has healed more and feels more comfortable in her body. (lesbian cisgender woman, 30y/7w, NBP)

The fact that the couple were talking about sex suggests an active process between them, aimed at creating change. Additionally, among those who had not engaged in sexual activity since childbirth, many described actively maintaining other forms of closeness:

Since we’ve been pregnant at the same time and now have two babies, sex is something that’s been prioritized the least, but it’s also something we’ve talked a lot about. We want intimacy and sex, and right now, step one is not forgetting to kiss each other, hug, etc. (lesbian cisgender woman, 32y, 37w and 12w, BP and NBP)

The excerpt shows a desire for more closeness and sex, which both partners appear to share. With two babies, the participant explained that sex was their “lowest priority.” Nevertheless, maintaining communication about intimacy seemed important for the couple during this period, possibly contributing to sustained emotional closeness—and to the longing for sex.

## Conclusion and implications for practitioners

The present study shows that LGBTQ+ couples in Sweden, to a large extent, experience similar sexual health challenges during pregnancy and the postpartum period as other couples. A decline in sexual desire was common and was associated with pregnancy- and birth-related complications as well as mental health issues. However, many couples described maintaining a close relationship through other means, prioritizing alternative forms of intimacy and settling into parenthood over sexual activity.

Some participants described how neither they nor their healthcare practitioners addressed sex or sexual health during their contacts. In addition, written information did not cover sex from an LGBTQ+ perspective. Sexuality thus appeared to remain an unspoken topic, with a lack of information influencing decisions about which sexual activities felt safe to engage in. For LGBTQ+ couples to feel comfortable addressing any thoughts, fears, or struggles they may have regarding sex during pregnancy and in the postpartum period, it is important that they are met with respect and affirmation by well-informed practitioners who do not hesitate to raise the subject themselves. While there is no evidence from the present study that LGBTQ+ individuals’ sexual health concerns would not be met with respect in Swedish perinatal care, their lack of information on safe sexual practices suggests that they may not have felt comfortable addressing the topic.

Lack of information about safe sexual activity during pregnancy and in the postpartum period can cause unnecessary stress for some LGBTQ+ individuals, sometimes leading them to avoid sex when not physically motivated. In these cases, adequate information is crucial. Taboos surrounding sex—or specific sexual activities—during the transition to parenthood may be alleviated when practitioners actively address the topic, affirming that various forms of sexual activity during pregnancy and postpartum are natural and can be enjoyable, as long as they are desired by all parties.

At an organizational level, it is important to ensure that healthcare professionals in perinatal care are supported through training in LGBTQ+ inclusive sexual health. Integrating content on affirming communication and sexual practices into existing education or continuing professional development may help practitioners address sexual health proactively and inclusively.

While the present study included data on LGBTQ+ couples in Sweden, it is important that LGBTQ+ couples’ sexual health during the transition to parenthood is addressed in further research, also including other national contexts.
